# Recombinant protein quality evaluation: proposal for a minimal information standard

**DOI:** 10.4056/sigs.1834511

**Published:** 2011-11-22

**Authors:** Ashley M. Buckle, Mark A. Bate, Steve Androulakis, Mario Cinquanta, Jerome Basquin, Fabien Bonneau, Deb K. Chatterjee, Davide Cittaro, Susanne Gräslund, Alicja Gruszka, Rebecca Page, Sabine Suppmann, Jun X. Wheeler, Deborah Agostini, Mike Taussig, Chris F. Taylor, Stephen P. Bottomley, Antonio Villaverde, Ario de Marco

**Affiliations:** 1 The Department of Biochemistry and Molecular Biology, School of Biomedical Sciences, Faculty of Medicine, Nursing and Health Sciences, Monash University, Australia; 2 Monash eResearch Centre, Monash University, Clayton, Victoria, Australia; 3 Cogentech, Protein Chemistry Unit, Milano, Italy; 4 Max Planck Institute of Biochemistry, Department of Structural Cell Biology, Martinsried, Germany; 5 Protein Expression Laboratory, SAIC-Frederick Inc., National Cancer Institute, Frederick, MD USA; 6 Structural Genomics Consortium, Karolinska Institutet, Department of Medical Biophysics and Biochemistry, Stockholm, Sweden; 7 IEO, Milano, Italy; 8 Brown University, Department of Molecular Biology, Cell Biology and Biochemistry, Providence, RI, USA; 9 Max Planck Institute of Biochemistry, Microchemistry Core Facility, Martinsried, Germany; 10 National Institute for Biological Standards and Control, Health Protection Agency, Hertfordshire, UK; 11 Protein Technologies Group, Babraham Bioscience Technologies, Cambridge UK; 12 The European Bioinformatics Institute, Wellcome Trust Genome Campus, Hinxton, Cambridgeshire, UK.; 13 Institute for Biotechnology and Biomedicine and Department of Genetics and Microbiology, Universitat Autònoma de Barcelona, and CIBER de Bioingeniería, Biomateriales y Nanomedicina (CIBER-BBN), Barcelona, Spain; 14 Department Environmental Sciences, University of Nova Gorica, Nova Gorica, Slovenia.; 15 Corresponding authors: Ario de Marco, University of Nova Gorica (UNG), Rožna Dolina (Nova Gorica), Slovenia. Tel. 0039.3493542056; ario.demarco@ung.si

## Presentation of the MIPFE checklist

A proposal for the introduction of the Minimal Information (MI) platform dedicated to the acquisition and annotation of data concerning recombinant proteins (Minimal Information for Protein Functionality Evaluation – MIPFE) was recently published [1] and discussed at the 5^th^ Recombinant Protein Production Conference (Alghero 2008) and the 2009 PEP Talk meeting (San Diego). The benefits of such standards are generally recognized, although there are concerns regarding its implementation as well as its perception of being too invasive for research freedom [2].

The meaning attributed to stored data is perceived differently within the MI community. The necessity of optimizing the quality of protein quality data annotation is generally acknowledged [3,4], since ontology and formal correctness are crucial for unambiguous data reporting and comparison, and ignoring such rules would decrease the accuracy of curation, lead to the loss of valuable information for efficient data mining and prevent the assessment of the experimental methods [5]. However, in certain domains further orthogonal corroboration of the same material used in reported experiments is highly desired for the identification and recognition of artifacts and assessment of the final results. For instance, it is still very often the case that published biological data are obtained with starting material, the structural characteristics of which have not been evaluated or made available [6]. As a result, there is a pressing need for good practice guidelines within publications and databases, as for example in the evaluation of the native state of proteins used for *in vitro* interaction assays [1,7].

The reputation of journals, as well as funding bodies, depends on data quality. However, data quality is often hard to evaluate during the peer-review process. This has not gone unnoticed in the editorial context, where, for example, improvements to the peer-review process have been suggested that will facilitate the collection, submission and validation of proteomic, microarray and, more recently, imaging data [8]. In addition, funding agencies are becoming increasingly concerned about the reliability and accessibility of data collected by laboratories which they fund [8-10]. We therefore argue that it is time to implement similar policies for the transparent and rigorous reporting of data in all publications concerning proteins. For example, it is often ignored that recombinant proteins form not only insoluble precipitates, but also soluble aggregates, mostly when carriers are fused to improve solubility [11-13]. Such aggregates may retain some function [13,14] and therefore, without controlled experiments aimed at defining monodispersity and native structure, the interpretation of experimental results is weakened. Thus, the scientific community (editors, reviewers, readers) must have access to the raw data to assess the biophysical characterization and, accordingly, be able to judge the quality of the proteins used in the experiments. Ideally, it will remain the responsibility of editors and referees to check the robustness of controls and, where necessary, to request further experiments using the original material. Integration of annotated control experiments into the main text offers a useful complementary evaluation tool for reviewers and readers. We consider that information concerning aggregation status and secondary structure should be reported as a minimal requirement for publication under Supplementary Material. These controls should be available when authors describe protein production as well as protein interaction experiments (pull-down, surface plasmon resonance, antibody/protein microarrays, and isothermal titration calorimetry).

In practice, it is important to define what is to be considered mandatory and what may remain optional within the MI package. An overly rigid and demanding protocol will be perceived as interference in the scientific work and most likely would be rejected by the community on these grounds. Recently, an interesting attempt at identifying a version of the MI guideline for describing proteins interacting in complexes has been reported [15]. However, it is difficult to judge the efficacy of the approach since the number of participants who volunteered to deposit the required information was limited to five.

In order to offer a workable solution for describing the MI for the evaluation of recombinant protein quality we propose a solution involving a repository to store the relevant results concerning protein construct features and biophysical characterization. Uploading of the information into the database is available through the MIPFE site [16]. We have designed a loosely structured text form allowing authors to describe the minimal information from an experiment which can be made available to reviewers, editors, and ultimately to other scientists. The proposed format requires little effort by the user (*e.g.* cut and paste using a simple text editor on any computing platform), and is human readable, yet sufficiently structured and formatted to allow data meta-analysis. Non-textual experimental results, such as gels and graphs, can be uploaded as image files alongside the form. In addition to its simplicity, the form can be copied and re-used by the authors and indeed the scientific community. Once deposited and validated, the dataset is given a unique handle which can be referred to in published manuscripts (for instance, as Supplementary Material), and possibly as a DOI tagged entity, as suggested recently [17].

Only the essential amount of obligatory information concerning the construct must be provided by the authors in the MIPFE form, in order to avoid possible misinterpretation  of any annotation [18,19]. The fields concerning characterization experiments remain optional and are intended as guidelines for controlled experiments that are run in order to evaluate protein structural quality.

Although our approach is designed to capture the minimal amount of data from the user as quickly and effortlessly as possible, the form does allow for raw data to be described and deposited, encouraging users to provide as complete an entry as possible. MI platforms evolve progressively to match needs and overcome limitations [20] and the logical future development of the one we propose could be the implementation of the MIBBI standardization guidelines for annotation [21,22], allowing more extensive annotation and ultimately data mining and bioinformatic analyses.
